# Differentiating the solution structures and stability of transthyretin tetramer complexed with tolcapone and tafamidis using SEC-SWAXS and NMR

**DOI:** 10.1107/S1600576725004716

**Published:** 2025-07-08

**Authors:** Orion Shih, Yu-Chen Feng, Sashank Agrawal, Kuei-Fen Liao, Yi-Qi Yeh, Je-Wei Chang, Tsyr-Yan Yu, U-Ser Jeng

**Affiliations:** a National Synchrotron Radiation Research Center Hsinchu300092 Taiwan; b Institute of Atomic and Molecular Sciences Academia Sinica Taipei106319 Taiwan; c Department of Chemistry National Taiwan Normal University Taipei11677 Taiwan; d Department of Chemical Engineering and College of Semiconductor Research National Tsing Hua University Hsinchu300044 Taiwan; European Molecular Biology Laboratory, Hamburg, Germany

**Keywords:** human transthyretin, tolcapone, tafamidis, solution structure, small- and wide-angle X-ray scattering, SWAXS, NMR

## Abstract

Human transthyretin (TTR) is a homotetrameric protein involved in transporting thyroxine (T4) and retinol-binding protein within serum and cerebrospinal fluid. The disassociation of TTR’s tetrameric structure can lead to the formation of biologically toxic TTR amyloid fibrils. Tolcapone, a small molecule currently under clinical trial, has shown potential as a TTR stabilizer and may act as an alternative to tafamidis, the conventional therapeutic agent used to prevent TTR dissociation. Using size-exclusion-chromatography-based small- and wide-angle X-ray scattering (SEC-SWAXS) complemented by nuclear magnetic resonance (NMR) spectroscopy, this study reveals the solution conformations of Apo-TTR and TTR complexed with tolcapone and tafamidis. Our results indicate that both compounds can bind similarly to the two T4 sites of TTR, leading to a small increase in the radius of gyration from 24.3 ± 0.1 Å (Apo-TTR) to 25.8 ± 0.1 Å. Consequently, both compounds largely stabilize the TTR against dissociation, denaturation and oligomerization up to 8 *M* urea, whereas Apo-TTR starts to denature at this concentration and forms larger oligomers at 8 *M* urea. Additionally, under a reduced TTR–drug mixing ratio of 1:1, which targets only one T4 site, tafamidis more effectively stabilizes the TTR tetrameric conformation at 8 *M* urea, a difference attributed to its higher affinity for the first T4 site. These results illustrate an effective strategy for investigating protein–drug interactions by examining the solution conformations of protein–drug complexes under physiological conditions, providing structural hints to the design of therapeutic agents targeting TTR.

## Abstract 2

The structures and stabilities of transthyretin tetramers and their complexes with tolcapone or tafamidis bound at the T4-site, in aqueous solutions without and with 8 *M* urea, are revealed using SEC-SWAXS and NMR.

## 1. Introduction

Human transthyretin (TTR) is a homotetrameric protein composed of two U-shaped dimer subunits that attach back to back, forming two identical funnel-shaped binding sites at the dimer–dimer interface (Yee *et al.*, 2019 ▸). TTR functions to transport thyroxine (T4) in the serum and cerebrospinal fluid through these binding sites (Pinheiro *et al.*, 2022 ▸). The sophisticated tetramer structure of TTR can disassociate, leading to the breakdown into monomers that are prone to aggregating into amyloid deposits. This aggregation results in transthyretin amyloidosis, a biologically toxic condition (Sant’Anna *et al.*, 2016 ▸; Yee *et al.*, 2019 ▸). The mechanism causing the destabilisation of the TTR tetramer is attributed to protein mutation, the origin of which, however, remains unclear (Liu *et al.*, 2019 ▸). One therapeutic approach involves stabilizing the tetramer structure through ligand binding at the T4 sites, which stabilizes TTR but may impair its T4-carrier function (Pinheiro *et al.*, 2022 ▸).

Tolcapone, originally developed in 1990s for the treatment of Parkinson’s disease, and tafamidis, an established treatment for transthyretin amyloidosis, are both recognized for their selective affinity for binding to the TTR T4-binding sites. Recently, tolcapone has been explored for its potential to stabilize TTR and prevent amyloid fibril formation. This interaction significantly stabilizes the tetrameric structure of TTR, which has been characterized through X-ray crystallography (Bulawa *et al.*, 2012 ▸; Sant’Anna *et al.*, 2016 ▸). According to a recent thermodynamic study (Pinheiro *et al.*, 2022 ▸), tolcapone binds more effectively to TTR than tafamidis on average, resulting in greater protein stabilization under specific conditions (Verona *et al.*, 2017 ▸). This enhanced performance of tolcapone is primarily attributed to its more enthalpically favorable interactions, particularly at the second T4-binding site, rendering tolcapone able to outperform tafamidis in terms of a full binding efficiency of the two T4 sites. Nevertheless, tafamidis exhibits significantly lower free energy of the first T4 site binding than tolcapone (Pinheiro *et al.*, 2022 ▸). Such differential binding affinity of tafamidis to the two T4 sites has practical advantages in balancing between ligand-stabilization and biological functions performed on the same T4 sites.

Currently, many crystal structures of the TTR tetramer complexed with ligands are documented in the literature (Bulawa *et al.*, 2012 ▸; Hamilton *et al.*, 1993 ▸, Sant’Anna *et al.*, 2016 ▸). However, subtle variations exist in these crystal structures, particularly in positioning specific loops and the side-chain rotamers at the T4-binding sites. These variations likely arise from differences in the crystallization environments. In practice, the static nature of crystallographic structures makes it challenging to ascertain the dynamic impacts of ligand binding on native TTR stability. To better understand the stabilizing effects of tolcapone and tafamidis on TTR tetrameric stability, studying solution structures of the ligand-complexed TTR under relevant environmental perturbations would be more revealing. Solution conformations can provide more kinetic insights into how the two compounds influence the stability and functional performance of TTR in physiological conditions.

In this study, we employ size-exclusion chromatography (SEC)-based small- and wide-angle X-ray scattering (SEC-SWAXS) along with nuclear magnetic resonance (NMR) spectroscopy to investigate the solution structural features of TTR and its complexes. Optical measurements of UV–Vis absorption and differential refractive index (dRI) are incorporated into the SEC-SWAXS measurements to assess the conformations in folding–unfolding and aggregation of TTR simultaneously (Shih *et al.*, 2023 ▸; Shiu *et al.*, 2008 ▸; Yeh *et al.*, 2017 ▸). SEC-SWAXS can separate the scattering contributions of the native tetramer, unfolded species and intermediate oligomers in a solution along the elution path for *in situ* separated SWAXS scattering that significantly simplifies the corresponding data analysis (Shih *et al.*, 2017 ▸).

Building on a previously developed protocol that integrates SWAXS with NMR data (Lin *et al.*, 2024 ▸), this study further incorporates molecular dynamics simulations using an explicit water model based on crystallographic structures to elucidate the solution conformations of Apo-transthyretin (Apo-TTR) and its complexes with tolcapone and tafamidis. Additionally, we assess the structural stability of TTR in the presence of these two pharmaceutical compounds incubated under high urea concentrations for several days. This integrated approach enables detailed insights into the conformational stability of TTR in solution and provides comparative evidence supporting the potential therapeutic efficacy of tolcapone and tafamidis under denaturing conditions.

## 2. Materials and methods

### 2.1. Recombinant TTR protein expression and purification

Recombinant wild-type (WT) TTR protein production and purification were carried out according to the description in the literature (Feng *et al.*, 2025 ▸; Liu *et al.*, 2019 ▸). Briefly, the synthetic codon-optimized WT *hTTR* DNA sequence was cloned into the pET21b expression vector, the B1 domain of Streptococcal protein G (GB1), followed by the 7x histidine tag and the cleavage site of tobacco etch virus (TEV) protease, at the N-terminal. The pET21b protein expression vector carrying the GB1–His7-tag–TEV–TTR sequence was transformed in BL21 (DE3) competent cells. WT TTR protein was overexpressed in *Escherichia coli* BL21 (DE3) in both LB and M9 minimal medium, prepared with [^15^N, 99%] ammonium chloride, by induction with 0.5 m*M* IPTG (Gold Biotechnology, USA) at 37°C for 6 h. The cells were re-suspended in lysis buffer (50 m*M* Tris, 300 m*M* NaCl, 10 m*M* imidazole, 10 m*M* β-ME pH 7.9) and lysed by a French press (Genizer, USA). The supernatant obtained after centrifugation at 15000 r.p.m. (JA 25.50 rotor, Beckman Coulter, USA) for 40 min at 4°C was purified using a nickel affinity column, pre-equilibrated with the equilibration buffer (50 m*M* Tris, 300 m*M* NaCl, 10 m*M* imidazole, 10 m*M* β-ME pH 7.9). GB1–His7-tag–TEV–TTR protein was finally eluted with an elution buffer (50 m*M* Tris, 300 m*M* NaCl, 250 m*M* imidazole, 10 m*M* β-ME pH 7.9). The protein was further purified using SEC of a column of HiLoad 16/600 Superdex 200 prep grade (GE Healthcare, USA), with a pH 7.9 buffer containing 50 m*M* Tris, 50 m*M* NaCl, 0.5 m*M* EDTA and 1 m*M* DTT. To remove the GB1–His-tag, TEV protease was added to the fused protein (GB1–TTR:TEV = 10:1) and incubated at 30°C for 72 h. The cleaved protein was then further purified by SEC using a HiLoad 16/600 Superdex 75 prep grade (GE Healthcare, USA).

### 2.2. NMR/SWAXS sample preparation

Three sets of [U-^15^N] labeled TTR sample solutions, without and with ligand (tafamidis or tolcapone), were prepared for 2D heteronuclear single quantum coherence (HSQC) NMR measurements to reveal amide proton–nitro­gen single-bond correlations. The uniformly ^15^N labeled WT TTR tetramer with a concentration of 125 µ*M* was first prepared in a buffer containing 50 m*M* NaPi, 50 m*M* NaCl, 0.5 m*M* EDTA and 1 m*M* TCEP pH 6.5. To prepare TTR ligand-binding sample solutions, 15 µl of either tafamidis or tolcapone DMSO solution of 10 m*M* was added to 300 µl of [U-^15^N] labeled TTR sample solution for mixtures of a molar ratio of 1:4 of TTR tetramer to the ligand. Subsequently, varying amounts of urea were added to the selected samples to achieve final urea concentrations ranging from 0 to 8 *M*, increasing in 1 *M* increments. All samples were incubated at room temperature for 2 days before HSQC spectrum collection at 25°C. In addition, the 2D ^1^H-^15^N transverse relaxation optimized spectroscopy (TROSY)-HSQC and 3D TROSY-HNCA spectra of 125 µ*M* [U-^15^N] labeled TTR in 5% DMSO, with and without tolcapone, were recorded at 30°C to analyze the chemical shift changes due to the ligand binding. The NMR sample buffer for this purpose contained an additional 5% of DMSO to completely dissolve tolcapone at a 2.5:1 molar ratio (tolcapone to TTR tetramer) to saturate the T4-binding sites of TTR. All these NMR experiments were recorded using an 800 MHz Bruker Advance III spectrometer equipped with a TCI probe.

Parallel TTR samples for NMR measurements were prepared for SWAXS measurements, with urea concentrations of 0 and 8 *M*, and the molar ratio of the ligand to TTR tetramer *R* = 0 or 10. Samples without and with urea were incubated at room temperature for at least 2–4 days before SWAXS measurements. Additional TTR samples with 8 *M* urea and different values of *R* = 1, 2 and 5 were prepared and incubated similarly.

### 2.3. SEC-SWAXS

SEC-SWAXS measurements were conducted at the TPS 13 A BioSWAXS endstation of the National Synchrotron Radiation Research Center, employing a 15 keV X-ray beam and two in-vacuum detectors, Eiger X 9M and X1M, for simultaneous SAXS and WAXS data collection, as described previously (Liu *et al.*, 2021 ▸; Shih *et al.*, 2022 ▸). A small amount (3 µl) of sample solution was first injected into the SEC-SWAXS system, comprising an in-line HPLC unit (Agilent 1260 series), UV–Vis absorption and refractive index spectrometers for initial sample evaluation. An Agilent Bio SEC-3 column with 300 Å pore size was used for all SEC-SAXS measurements with a sample injection volume of 10 µl (for better-separated elution peaks and zero-angle scattering intensity *I*_0_ values) or ∼100 µl (for better high-*q* data quality). The sample elution was then directed to a quartz capillary (2 mm diameter, 10 µm wall thickness) for concurrent X-ray and UV–Vis exposure (2 s per frame rate over the sample elution peak), followed by dRI measurements approximately 40 cm downstream. The dRI profile was corrected for sample diffusion broadening along the additional 40 cm sample elution pathway to overlap the online UV–Vis absorption profile measured (using a TIDAS MCS UV–NIR spectrometer positioned perpendicularly to the X-ray beam) at the same sample capillary position as for SAXS (Shih *et al.*, 2022 ▸).

The extinction coefficient of TTR at 280 nm, 74050 *M*^−1^ cm^−1^ in water, was used to calculate the protein concentration based on the UV absorbance measured along the sample elution, using the Beer–Lambert law. The deduced sample concentrations are consistent with that obtained using the concomitantly measured dRI data based on a refractive index increment value of d*n*/d*c* = 0.187 ml g^−1^ (at 633 nm wavelength) for human proteins larger than 10 kDa (Zhao *et al.*, 2011 ▸). The band broadening process for the alignment of dRI and UV–Vis absorbance profiles was carried out with the *ASTRA* software (Wyatt Technology), performing a combined analysis of the concomitantly measured dRI and UV–Vis absorption data.

In addition to the 2 s per frame SAXS data collected during the sample elution peak, 3–4 data frames for buffer scattering (each of 40 s exposure time) were collected both before and after the elution peak, and selectively used in background subtraction according to the sample exposure condition. Buffer scattering prior and close to the sample elution peak was used in the SWAXS background subtraction. Post-sample buffer scattering might be used in case of capillary contamination from sample radiation damage. For the sample solutions with excess ligands, scattering background subtraction was done with the buffer solution containing no ligands. During the SEC-SWAXS measurements, the autosampler chamber, the SEC column and the sample capillary cell were thermostated at either 299 or 310 K (26 or 37°C) (Shih *et al.*, 2022 ▸). With the TPS 13A SWAXS *Data Reduction Kit* (*DRK*) (version 3.6), overlapping SAXS profiles showing no sign of radiation damage (indicated by increasingly up-turned low-*q* intensity during irradiation) or concentration-dependent effects (decreased low-*q* intensity with increasing concentration) were selected, merged and then corrected for buffer scattering. After normalizations with incoming X-ray flux and sample thickness, the merged data were rescaled to absolute scattering intensity (in cm^−1^) by calibrating against the absolute scattering intensity of water.

Each SAXS data frame over the elution peak was evaluated using the TPS-13A *DRK*, which integrates several *ATSAS* functions for data evaluation (Manalastas-Cantos *et al.*, 2021 ▸; Franke *et al.*, 2025 ▸), including examinations of the radius of gyration *R*_g_ for monodispersity and Kratky plots for protein unfolding tendency. The SAXS zero-angle X-ray scattering intensity *I*_0_ and *R*_g_ values were retrieved, using the Guinier approximation, across the elution peak to assess the monodispersity and overlapping of the collected SAXS data frames. Only the frames within the elution peak that showed stable *R*_g_ values and well overlapped SAXS profiles were selected and merged to calculate the distance distribution function *p*(*r*), which was generated for further data quality examination using *GNOM* (Svergun, 1992 ▸). Corresponding WAXS data frames were also processed collectively using the *DRK* software. Both data sets were then merged for SWAXS data of the broad scattering vector magnitude *q* range 0.008–2.0 Å^−1^, with *q* = 4πλ^−1^sinθ defined by the X-ray wavelength λ and the scattering angle 2θ.

The concomitantly measured elution profiles of UV absorbance and the SAXS *I*_0_ were used to extract the aggregation number *N* (= 4 for tetramer) over the elution peak using *N* = (*I*_0_/*C*)/*I*_0*m*/*c*_. Here, *I*_0_ values were extrapolated from the SAXS profile using the Guinier approximation, and *C* is the TTR monomer concentration derived from the concomitantly measured UV intensity at 280 nm (UV_280_); *I*_0*m*/*c*_ is the *I*_0_ value per unit monomer concentration (mg ml^−1^) calculated from the contrast of the scattering length density of the TTR monomer with respect to the buffer solution used.

### 2.4. SWAXS data analysis

In a previous report (Tsai *et al.*, 2023 ▸), we established a protocol based on the *Rosetta* modeling suite (Das & Baker, 2008 ▸; Kaufmann *et al.*, 2010 ▸; Bender *et al.*, 2016 ▸) to construct all-atom models of small non-compact proteins under the constraint of the SAXS data fitting χ^2^ to the model (Stovgaard *et al.*, 2010 ▸). However, the structures of the crystalline forms of Apo-TTR (PDB entry 1tta; Hamilton *et al.*, 1993 ▸) and tolcapone-bound TTR (Sant’Anna *et al.*, 2016 ▸) are too large to be refined using the *Rosetta-fastsaxs* protocol with the SWAXS data. As an alternative, *CRYSOL* (Franke *et al.*, 2017 ▸; Svergun *et al.*, 1995 ▸) is used for structure comparison purposes. *CRYSOL* calculates the SWAXS profile of a crystal structure with an added water shell; with the setting of implicit hydrogens, *CRYSOL* disregards the hydrogens of the input PDB file. We note that the measured SWAXS data include the bound water scattering contribution in the high-*q* region (above ∼1 Å^−1^) which may not be accounted for adequately by the implicit water shell used in *CRYSOL*. With the structural sensitivity of SEC-SWAXS covering a wide *q* range, the hydration water structures of biomolecules become relevant in the SWAXS data analysis, as demonstrated recently (Knight & Hub, 2015 ▸; Hub, 2018 ▸; Schroer & Svergun, 2018 ▸; Manalastas-Cantos *et al.*, 2021 ▸; Shiu *et al.*, 2025 ▸). To better account for the scattering contribution of the bound water molecules in the high-*q* region, we also computed the SWAXS profiles of the TTR crystal structures with *WAXSis*. The web server *WAXSis* computes SWAXS curves based on all-atom molecular dynamics simulations, using the explicit hydration water model TIP3P and the force field AMBER03 (Knight & Hub, 2015 ▸).

## 3. Result and discussion

### 3.1. Solution structure of TTR

**Figure 1:**
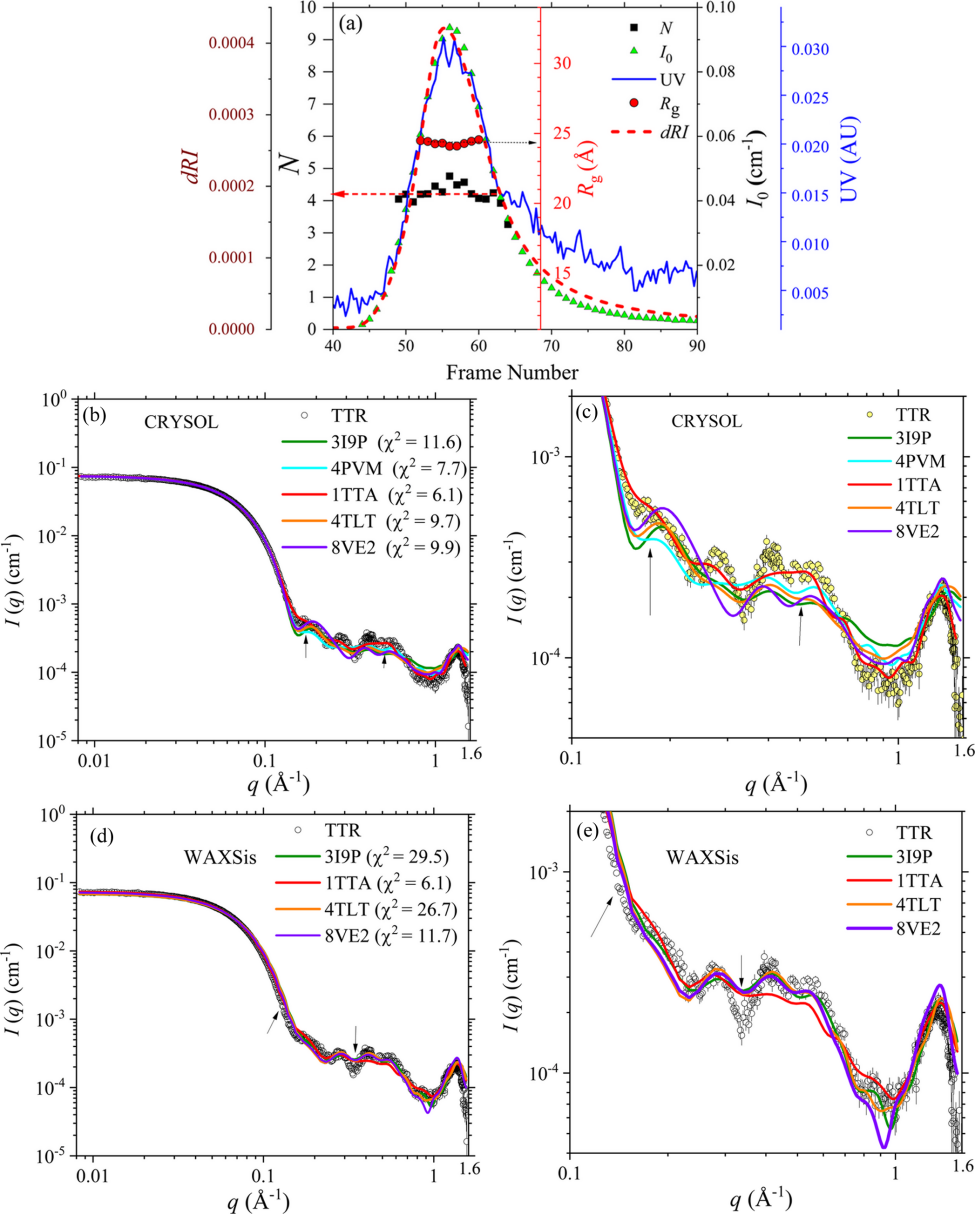
(*a*) Elution profiles of the UV_280_, dRI, and SAXS *I*_0_, *R*_g_ and aggregation number *N* (= 4 for tetramer) of the native TTR over the SEC-SWAXS elution peak. The frame number corresponds to the SWAXS data collection interval (2 s per frame) which was initiated selectively, slightly ahead of the onset of the sample elution peak. (*b*) Global and (*c*) local comparisons of the SWAXS data (SASBDB entry code SASDXW3) with the calculated SWAXS profiles (with the χ^2^ shown) of the existing TTR crystal structures (PDB codes indicated) using *CRYSOL* (version 3.0). (*d*) and (*e*) Similar comparisons with the SWAXS profiles calculated using *WAXSis*. Arrows in (*b*), (*c*), (*d*) and (*e*) mark the regions of more prominent fitting deviations.

Fig. 1 ▸(*a*) shows the SEC-SWAXS elution profiles of native TTR, including the measured *I*_0_ and UV–Vis optical density. The largely constant value of *R*_g_ = 24.3 ± 0.1 Å extracted from the SWAXS data across the elution peak indicates a single species. Furthermore, using the concomitantly measured UV absorbance and *I*_0_, we could deduce *N* ≃ 4 over the elution peak [Fig. 1 ▸(*a*)], confirming the observed monodisperse species to be the TTR tetramer (as detailed in the supporting information).

The corresponding SWAXS profiles [Small Angle Scattering Biological Data bank (SASBDB; https://www.sasbdb.org/) entry code SASDXW3] are fitted using a few often-referenced crystal structures of TTR [Fig. 1 ▸(*b*)] using *CRYSOL*. The calculated SWAXS profiles overlap the SWAXS data in the *q* range below 0.1 Å^−1^, indicating that these crystal structures share nearly the same size and shape (Fig. 1 ▸). Nevertheless, the crystal structure 1tta of human native TTR (Groenning *et al.*, 2015 ▸; Hamilton *et al.*, 1993 ▸) most closely describes the overall SEC-SWAXS data features up to *q* = 1.6 Å^−1^, compared with those calculated with other crystal structures with the PDB codes 3i9p (Lima & Foguel, 2009 ▸), 4pvm (Haupt *et al.*, 2014 ▸), 4tlt (Saelices *et al.*, 2015 ▸) and 8ve2 (Basanta *et al.*, 2025 ▸). The SWAXS profiles calculated from these crystal structures exhibit minor but differentiable variations in the higher-*q* region >0.1 Å^−1^, as shown in Fig. 1 ▸(*c*), revealing subtle local structural differences. We note that these TTR crystals were crystallized under different conditions, as summarized in Table 1 ▸. Likely, the protein structures were subject to different packing forces in the crystals, resulting in the local structure differences revealed, reflecting their different crystallization environments. A similar crystal packing force was also discussed in previous reports (Bertini *et al.*, 2009 ▸; Schirò *et al.*, 2020 ▸). In general, the trend of fitting χ^2^ summarized in Table 2 ▸ supports the notion that crystal structures with higher solvent content tend to better reproduce the solution SWAXS data, consistent with the previous reports. There are, however, exceptions reflecting additional factors directly from the differences between the protein crystal and solution structures beyond solvent content, which can influence the fit quality.

To more accurately assess the scattering contributions from hydration water molecules around TTR, we also calculated the SWAXS profiles with the same crystal structures using *WAXSis*, which incorporates an explicit solvent model, as previously described. The resulting profiles and χ^2^ values from both *WAXSis* and *CRYSOL* simulations are largely comparable, indicating that the implicit solvent model used in *CRYSOL* adequately approximates the protein scattering in solution up to the observed high-*q* region. However, *WAXSis* demonstrates improved fits to the experimental SWAXS data in the high-*q* region [Fig. 1 ▸(*e*)], revealing the advantage of using *WAXSis* with the explicit water model in capturing hydration water structures.

### 3.2. Binding structure of TTR with the ligands

**Figure 2:**
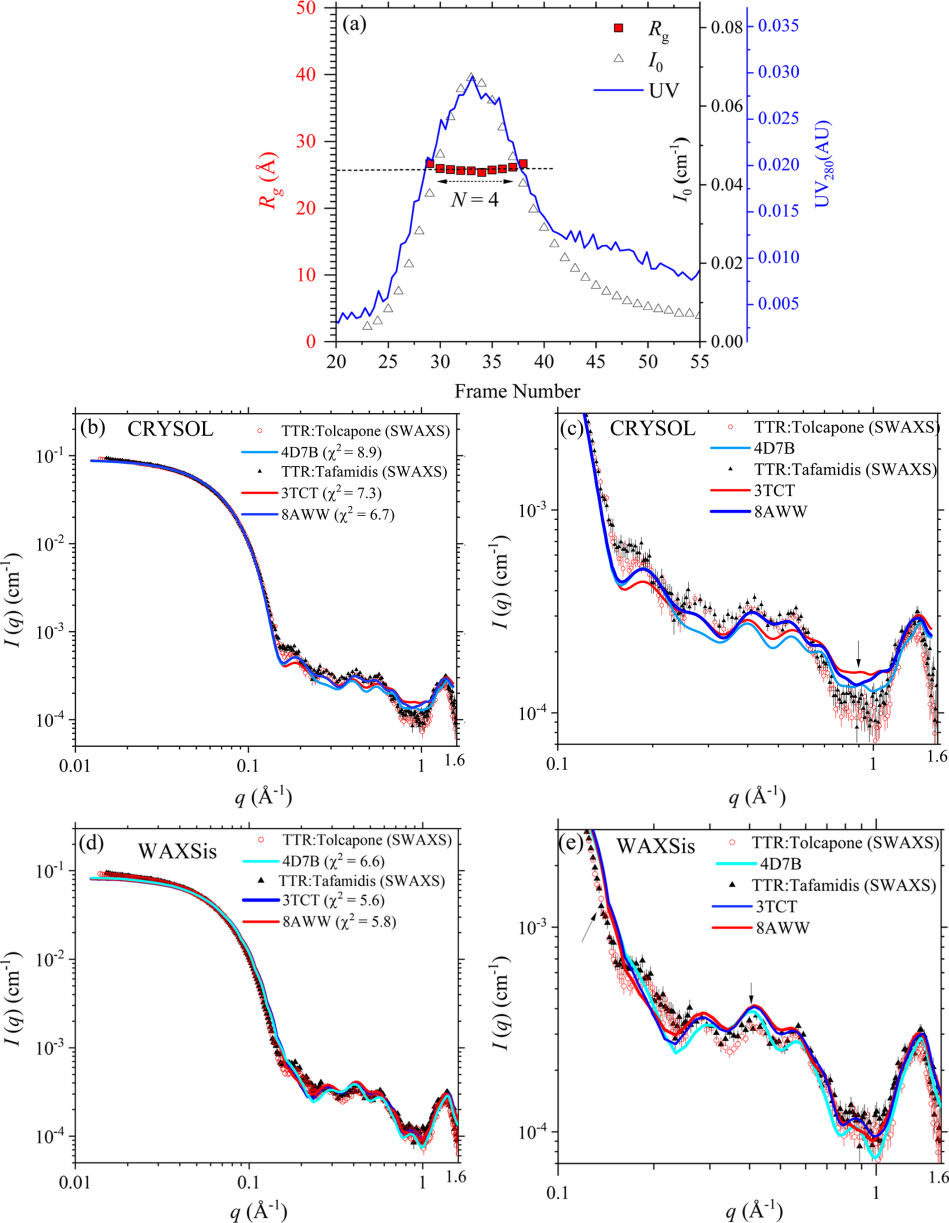
(*a*) Elution profiles of UV_280_ and SAXS *I*_0_ and *R*_g_ over the elution peak of the sample solution of TTR–tolcapone (1:10). The arrow indicates the region of the tetramer (*N* = 4). (*b*) Global and (*c*) local comparison of the SWAXS profiles of the TTR solutions, with tolcapone and tafamidis (*R* = 10) (SASBDB entry codes SASDXX3 and SASDXY3). The data are fitted (with the χ^2^ indicated) using *CRYSOL* with the crystal structures 3tct and 8aww of TTR–tafamidis and 4d7b of TTR–tolcapone. (*d*) and (*e*) Same sets of SWAXS data fitted using *WAXSis*. Arrows in (*c*) and (*e*) mark the regions of larger fitting deviations.

We measured SEC-SWAXS for the respective ligand–TTR solutions of oversaturated tolcapone [Fig. 2 ▸(*a*)] and tafamidis, with a ligand:TTR molar ratio *R* = 10 (SASBDB entry code SASDXX3 for tolcapone–TTR and SASDXY3 for tafamidis–TTR). The high *R* ratio used is to ensure full binding of the two ligands to the two T4 sites, as suggested in a previous report (Pinheiro *et al.*, 2022 ▸). The two SWAXS profiles [Fig. 2 ▸(*b*)] overlap well in the wide *q* range. This suggests very similar ligand-binding solution conformations of TTR with these two complexes, having the same *R*_g_ value of 25.2 ± 0.1 Å, which is only slightly larger than that (24.3 ± 0.1 Å) of the native TTR (Fig. 1 ▸). Moreover, the SWAXS profiles of TTR without or with the two ligand bindings overlap well in the wide *q* range as illustrated in Fig. S2, revealing that both tafamidis and tolcapone can bind to the same T4-site pockets with high similarity, without much disturbance of the TTR structural features in solution.

Using *CRYSOL* and *WAXSis*, we fit the two sets of SWAXS data with the corresponding crystal structures 3tct (Bulawa *et al.*, 2012 ▸) and 8aww (Cerofolini *et al.*, 2023 ▸) of the tafamidis–TTR complex and 4d7b (Sant’Anna *et al.*, 2016 ▸) of the tolcapone–TTR complex, respectively. The results shown in Fig. 2 ▸ illustrate that the full SWAXS data of the tafamidis–TTR complex can be comparably well fitted by the 3tct and 8aww structures, whereas the 4d7b crystal structure for TTR–tolcapone exhibits larger deviations from the SWAXS data in the higher-*q* region (0.3–0.7 Å^−1^). By contrast, with the explicit water model TIP3P, *WAXSis* can improve all the SWAXS data fitting (with reduced χ^2^) with the three crystal structures, particularly in the higher-*q* region as shown in Figs. 2 ▸(*d*) and 2 ▸(*e*). Likely, the ligand binding (either tafamidis or tolcapone) to the two T4 sites of TTR traps water molecules between the two T4 sites, leading to the higher scattering contributions [Fig. 2 ▸(*e*)] revealed by the explicit water model of *WAXSis*.

**Figure 3:**
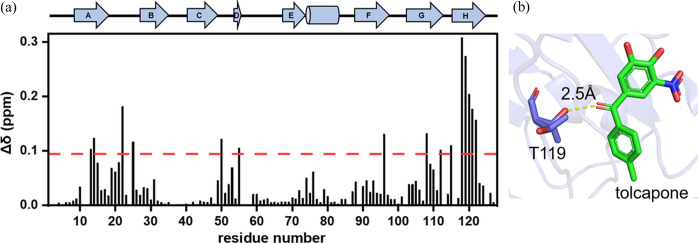
(*a*) NMR characterizations of tolcapone binding to TTR, with the residue-specific chemical shift changes Δδ of TTR on binding to tolcapone higher than the red dashed line (the mean value plus one standard deviation of Δδ). (*b*) Close-up view of the binding pocket T4 site showing the key hydrogen bonding amino acids involved in the coordination of tolcapone with TTR.

We further examined the TTR–tolcapone complex using TROSY-HSQC spectra. As shown in Fig. 3 ▸(*a*) the normalized chemical shift perturbation of TTR due to tolcapone binding was calculated according to (Williamson *et al.*, 1997 ▸)
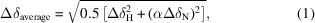
where Δδ_H_ and Δδ_N_ are the chemical shift change in ^1^H and ^15^N dimensions, and α is a weighting factor set to 0.2 for glycine and 0.14 for the rest of the amino acids. The normalized chemical shift perturbation was 0.04 p.p.m. with a standard deviation of 0.05 p.p.m. The chemical shift perturbations of the residues near the N and C termini are most prominent, including M13, V14, G22, A25, S50, L55, T96, A108, S112, S115, T118, T119, A120, V121 and V122. These residues are mainly located in the T4 sites of the dimer–dimer interface of TTR defined previously, thereby stabilizing the dimer–dimer interfaces. The crystal structure of the TTR–tolcapone complex (PDB entry 4d7b) reported previously (Madej *et al.*, 2014 ▸) further elucidates a key hydrogen bonding of the side-chain OH group of T119 with the ketone group of tolcapone in the T4 site, as illustrated in Fig. 3 ▸(*b*). In our previous report, we also found (with solution NMR) that tafamidis binds to WT TTR (Liu *et al.*, 2019 ▸) at the same T4 site but with a different origin of halogen bonding with the chloride moiety. These observations are consistent with those reported in a recent solid-state NMR analysis (Cerofolini *et al.*, 2023 ▸) and the X-ray crystal structure of the complex revealed previously (Bulawa *et al.*, 2012 ▸; Pinheiro *et al.*, 2022 ▸).

### 3.3. Urea-induced unfolding of TTR with both T4 sites bound with ligands

#### 3.3.1. NMR results

**Figure 4:**
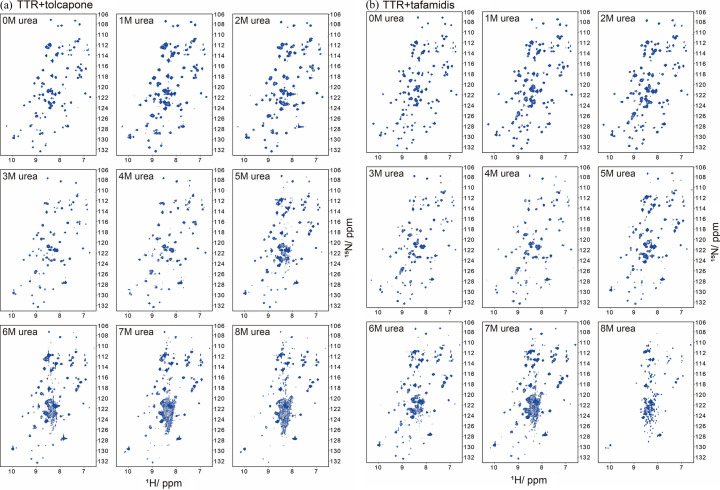
2D [^1^H,^15^N]TROSY-HSQC spectra of TTR with (*a*) tolcapone and (*b*) tafamidis binding under different urea concentrations at 25°C.

The ligand-binding enhanced structural stabilization of TTR was further examined under urea-induced unfolding by solution NMR spectroscopy. The 2D [^1^H,^15^N]TROSY-HSQC spectra of Apo-TTR under different urea concentrations are shown in Fig. S1 of the supporting information. At 3 *M* urea, a few new correlation peaks were observed at the center of the TROSY-HSQC spectrum, indicating initiation of the urea denaturation. At 5 *M* urea, there were no detectable TROSY-HSQC cross peaks corresponding to the native conformation of TTR. The TROSY-HSQC spectral pattern gradually changes, and the cross peaks are highly merged to a central zone at 8 *M*, revealing a largely unfolded structure (Fig. S1). In contrast, the TROSY-HSQC spectra of both tolcapone- and tafamidis-bound TTR still maintain similar patterns to the native TTR (0 urea) up to 5 *M* urea, as shown in Fig. 4 ▸. Above 5 *M* urea, peak broadening emerges, resulting in a smeared spectral pattern; however, TROSY-HSQC cross peaks corresponding to the native conformation of TTR are still detectable. The smear spectral pattern develops slowly with the increase of urea concentrations from 6 to 8 *M*, and the spectral patterns maintain a certain clarity up to 8 *M* urea, with respect to that of the unfolded Apo form. These results reveal that these two ligands can have comparable stabilizing effects on the TTR tetrameric structure.

#### 3.3.2. SEC-SWAXS result

**Figure 5:**
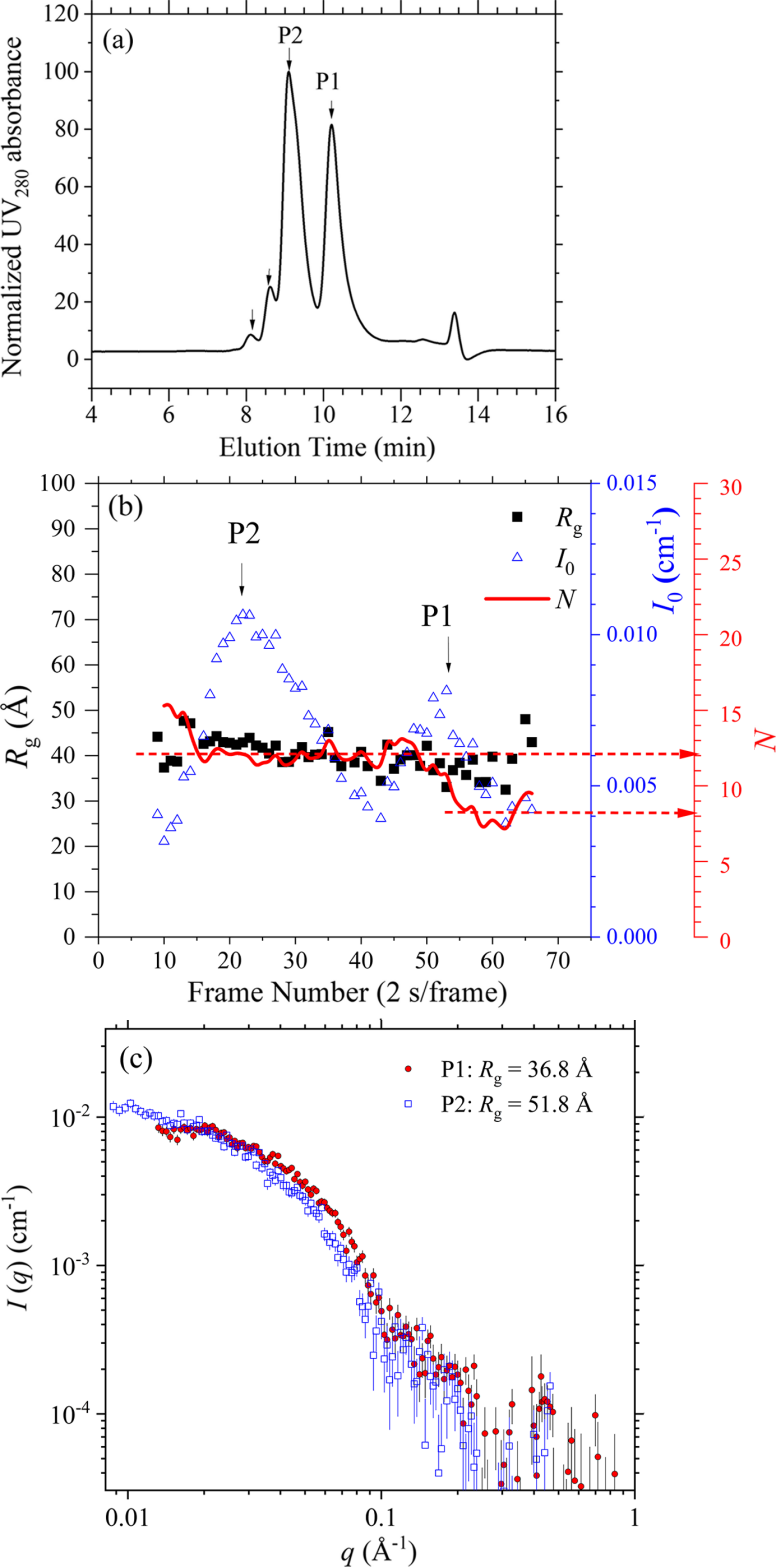
(*a*) Independent SEC elution profile (3 µl injection) of UV_280_ for TTR in 8 *M* urea at 37°C, showing multiple peaks (indicated by arrows) of TTR oligomers. (*b*) SEC-SAXS elution profiles (10 µl injection) of *I*_0_, *R*_g,_ and aggregation number *N* deduced over the two major elution peaks P1 and P2 in (*a*). (*c*) Corresponding SWAXS profiles of the same two major species, P1 and P2 of Apo-TTR, with the *R*_g_ values indicated.

We further examine the corresponding conformational changes of Apo-TTR in urea-induced unfolding using SEC-SWAXS. Consistent with the HSQC NMR characterization, we found that Apo-TTR can sustain its tetramer structure (*N* = 4) up to 4 *M* urea, with a marginal increase in *R*_g_ by *ca* 1 Å (Fig. S3). With 8 *M* urea, multiple peaks in the elution profile of the UV–Vis spectrum could be observed [Fig. 5 ▸(*a*)]. These discrete peaks suggest the formation of oligomers with specific aggregation numbers, which is consistent with a previous observation (Dasari *et al.*, 2019 ▸). The two major peaks of the SEC-SWAXS elution [Fig. 5 ▸(*b*)] correspond to two distinct species of *R*_g_ = 51.8 and 36.8 Å. With the correlated UV–Vis and *I*_0_ data measured, we could deduce the aggregation numbers *N* = 12 and *N* = 8 for the two species [Fig. 5 ▸(*b*)]. Therefore, we assign the elution peaks P1 as di-tetramer (*N* = 8) and P2 as tri-tetramer (*N* = 12). Moreover, the corresponding SWAXS profiles [Fig. 5 ▸(*c*)] exhibit significantly decayed features in the high-*q* region, suggesting that the local structures of these oligomers are severely distorted or unfolded compared with the native tetramer.

**Figure 6:**
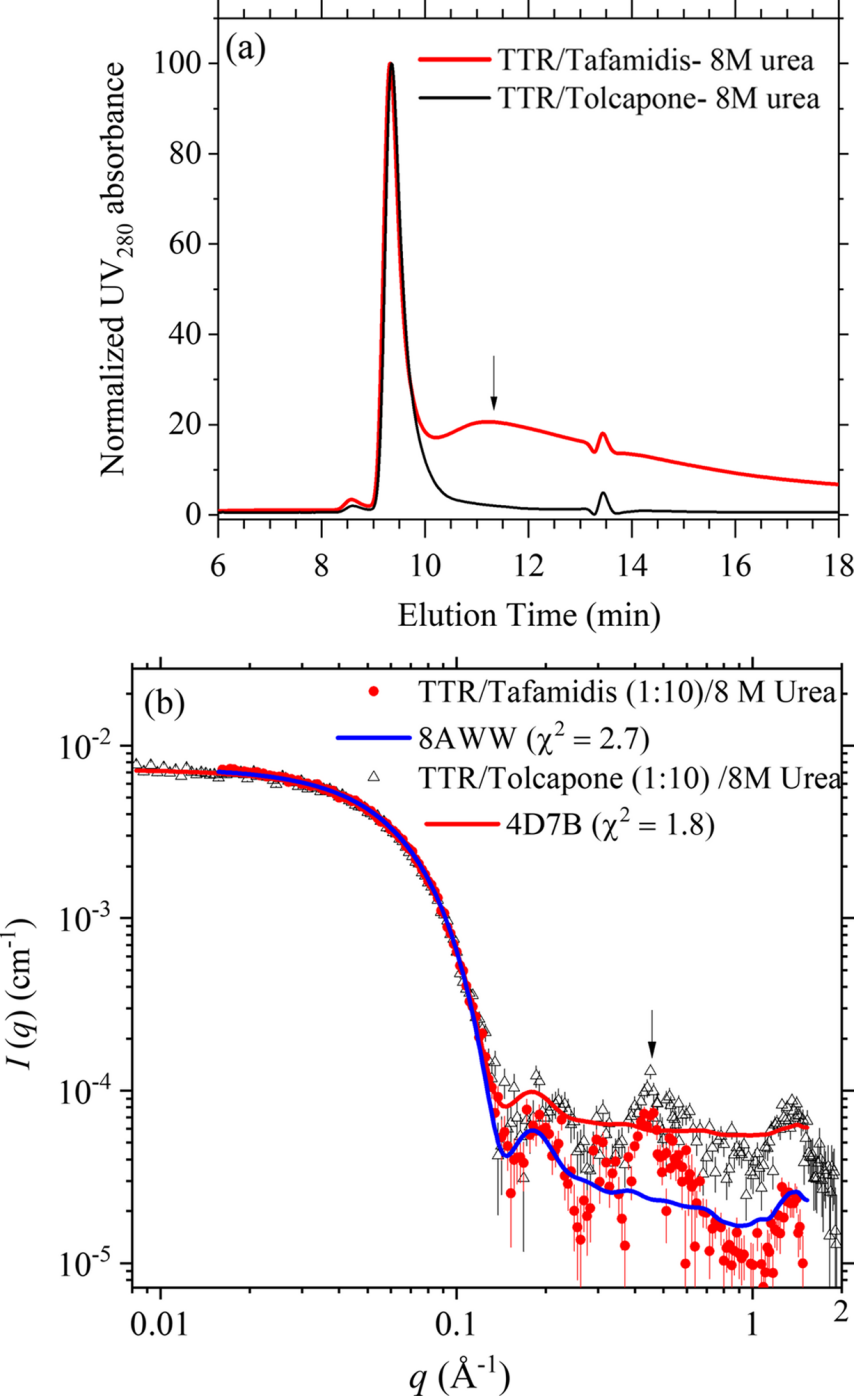
(*a*) UV_280_ intensity evolution profiles of the SEC-SWAXS of TTR–tafamidis and TTR–tolcapone solutions (*R* = 10), containing 8 *M* urea at 37°C. The arrow indicates a broad band that is likely contributed by dissociated TTR fractures in the former case. (*b*) Corresponding SWAXS profiles over the SEC-SWAXS elution peak. The calculated SWAXS profile of the 4d7b crystal structure of TTR–tafamidis largely overlaps with the SWAXS data, especially in the low-*q* region. Note that in Fig. 5 ▸(*a*) the elution time of the P1 peak assigned to the TTR octamer falls behind the elution peak of the smaller TTR tetramer in (*a*). The longer elution time is attributed to the unfolded/extended protein conformation in the TTR octamer, as similarly observed previously in the elution of unfolded bovine serum albumin (Yeh *et al.*, 2017 ▸) shown in Fig. S7.

At 8 *M* urea, the elution profiles of the UV–Vis spectra of the solutions of TTR–tolcapone and TTR–tafamidis both still maintain a monodisperse peak [Fig. 6 ▸(*a*)]; the latter case with tafamidis, however, reveals a broad band after the main peak, suggesting some of the TTR tetramer might dissociate. Fig. 6 ▸(*b*) shows the corresponding SWAXS profiles obtained over the elution peak, consistently revealing largely overlapped SWAXS profiles of similarly unperturbed conformation. The corresponding *R*_g_ values of TTR–tafamidis (*R*_g_ = 26.8 ± 0.5 Å) and TTR–tolcapone (*R*_g_ = 26.0 ± 0.5 Å) are about the same, and both are marginally increased from 25.2 Å of the TTR–ligand in solution without urea. These results indicate that tafa­midis and tolcapone have comparably good TTR-stabilization effects.

Note that the large scattering intensity fluctuations observed in the high-*q* region of Fig. 6 ▸(*b*) primarily result from the significantly reduced scattering contrast of the protein in the presence of 8 *M* urea. The low contrast leads to weaker scattering intensity. This makes background subtraction more challenging, particularly due to the additional scattering contributions from excess ligands near the sample elution peak, which becomes more pronounced in the high-*q* range. Despite these complications, key scattering features remain identifiable and comparable to those observed under urea-free conditions (Fig. 2 ▸). Reducing the ligand-to-protein molar ratio from *R* = 10 to *R* = 2 significantly alleviates the excess ligand scattering issue. Consequently, the SWAXS profiles of the TTR–ligand complex in 8 *M* urea, with *R* = 2, closely resemble those obtained without urea (Fig. S4), with nearly the same *R*_g_ values of 25.8 ± 0.1 Å for TTR–tafamidis and 25.7 ± 0.1 Å for TTR–tolcapone. These results further support that both ligands bind highly effectively to the two T4 sites of TTR and retain comparable stabilizing effects under the 8 *M* denaturing condition over a 4 day incubation at room temperature.

The combined effects of temperature and incubation time on the structural stability of three TTR sample solutions in 8 *M* urea were also investigated, specifically, native TTR and TTR complexed with either tafamidis or tolcapone (at a molar ratio *R* = 10). The samples were incubated at 4°C for 2 days and at room temperature for 4 days. As shown in Fig. S5, native TTR without ligands exhibited enhanced dissociation and aggregation under the prolonged incubation at elevated temperature. In contrast, both the tafamidis- and tolcapone-bound TTR samples demonstrated comparable stabilization effects, with only a minor increase in *R*_g_ of approximately 1 Å in the more stringent incubation condition.

### 3.4. Urea-induced unfolding of TTR with the first T4 sites bound with ligand

**Figure 7:**
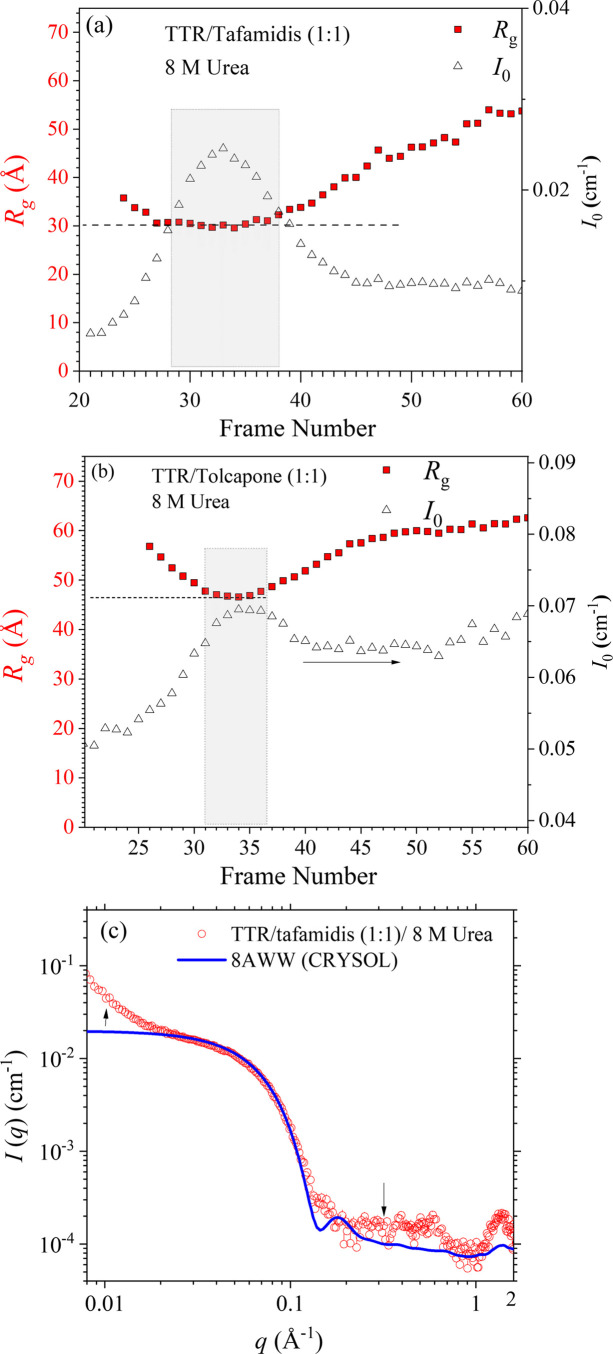
(*a*) SEC-SWAXS elution profiles of SAXS *I*_0_ and *R*_g_ for TTR with tafamidis at the binding ratio *R* = 1, in an incubated sample solution of 8 *M* urea, measured at 37°C. (*b*) Parallel result for TTR–tolcapone. (*c*) Corresponding SEC-SWAXS profile of TTR–tafamidis (1:1). For comparison, the SWAXS profile calculated using *CRYSOL* with the crystal structure 4d7b of tafamidis–TTR is also shown (solid curve).

Previous research by Pinheiro *et al.* (2022 ▸) highlighted the differentiable binding affinities of tafamidis and tolcapone to the two T4 sites of TTR. Tafamidis showed distinct binding free energy levels to the two T4 sites, lower at the first and higher at the second, unlike tolcapone’s consistent and intermediate binding energy across both sites. Namely, tafamidis exhibits a higher T4-site binding efficiency when the ligand-to-TTR molar ratio in solution is below 1:1. To further explore the differential T4-site binding effects on TTR stability, we conducted SEC-SWAXS analysis at a ligand–TTR mixing ratio of 1:1, confining ligand binding primarily to the first T4 site. The SEC-SWAXS results shown in Fig. 7 ▸(*a*) indicate that the TTR–tafamidis (1:1) complex essentially maintains a predominant elution peak of the tetramer. The *R*_g_ value, however, increases to 30 Å in the 8 *M* urea solution, compared with that (25.2 Å) without urea.

The SWAXS profile of the TTR–tafamidis complex [Fig. 7 ▸(*c*)], extracted from the elution peak within the region of constant *R*_g_, can still be partially fitted using the crystal structure of the TTR–tafamidis complex (PDB entry 4d7b). This partial agreement suggests that certain structural features of the tetrameric complex persist in 8 *M* urea, albeit to a limited extent. However, the significantly elevated scattering intensity observed in the low-*q* region, deviating from the fitted curve, indicates additional scattering contributions from a fraction of unfolded and/or aggregated TTR species present in the solution. In contrast, the corresponding TTR–tolcapone (1:1) sample [Fig. 7 ▸(*b*)] displays a broadened UV–Vis elution profile and a markedly increased *R*_g_ range (47–60 Å), indicating pronounced TTR aggregation. These results underscore the conditionally better stabilizing effect of tafamidis over tolcapone when the ligand-to-TTR molar ratio is below 1. This suggests that tafamidis may be favorable for long-term therapeutic efficacy in maintaining TTR tetramer integrity under clinical conditions where drug concentrations decline over time.

## 4. Conclusions

On the basis of integrated analyses using SEC-SWAXS, NMR, optical spectroscopies, and complementary SWAXS simulations of TTR crystal structures with both implicit and explicit hydration models, we elucidate the solution conformations of transthyretin and its complexes with ligands bound at the two thyroxine-binding sites along the dimer–dimer interface. When both T4 sites are fully occupied by either tolcapone or tafamidis, the resulting TTR–ligand complexes exhibit similarly enhanced tetrameric stability against urea-induced denaturation, maintaining structural integrity even in 8 *M* urea at 37°C after four days of incubation at room temperature. However, under partial binding conditions using a 1:1 ligand-to-TTR molar ratio in 8 *M* urea, tafamidis displays superior stabilization effects, attributed to its higher binding affinity for the first T4 site compared with tolcapone. These observations highlight the significance of differential site binding in modulating TTR stability and suggest a promising direction for next-generation TTR-stabilizing drug design. Rather than solely maximizing binding affinity at both T4 sites, a strategic focus on optimizing primary site binding while attenuating secondary site interaction may offer a refined approach, preserving TTR’s native conformation while minimizing potential interference with its physiological roles.

## 5. Related literature

The following additional reference is cited in the supporting information: Trewhella *et al.* (2017 ▸).

## Supplementary Material

Supporting figures and table. DOI: 10.1107/S1600576725004716/vg5150sup1.pdf

## Figures and Tables

**Table 1:** Summary of TTR samples for the SWAXS and NMR experiment, with samples incubated at room temperature for 4 days (SWAXS) or 2 days (NMR) Buffer solutions of SWAXS samples include 20 m*M* Tris and 50 m*M* NaCl; for NMR, 50 m*M* NaPi, 50 m*M* NaCl, 0.5 m*M* EDTA, 1 m*M* TCEP and 6% D_2_O were used. Note that 299 K was also used for the SWAXS samples with the urea concentration *C*_urea_ = 8 *M* and a ligand:TTR tetramer ratio of *R* = 10.

Experiment	Ligand:TTR ratio *R*	*C*_urea_ (*M*)	pH	Sample *T* (K)
SWAXS	*R* = 0	0 and 8	7	310
	Tafamidis (*R* = 1, 2, 5, 10)	8	7	310
	Tolcapone (*R* = 1, 2, 5, 10)	8	7	310

NMR	*R* = 0	0–8	6.5	298
	Tafamidis (*R* = 4)	0–8	6.5	298
	Tolcapone (*R* = 4)	0–8	6.5	298

**Table 2:** Summary of the reference X-ray and neutron (4pvm) crystal structures of TTR, including their crystallization conditions, 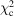 and 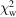 values, from data fittings with *CRYSOL* and *WAXSis*, respectively, and values of the Matthews coefficient *V*_m_ (defined as the crystal volume per unit of protein molecular weight, which is proportional to the solvent content in the crystal) adopted from the corresponding PDB entries, in the presence and absence of the two ligands of tafamidis and tolcapone Note that the 4pvm structure is unstable in a *WAXSis* simulation, yielding no χ^2^ value.

PDB entry	Ligand	Crystallization conditions	pH	Temperature (K)	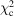 / 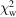	*V*_m_
3i9p	None	28% PEG 400, 0.2 calcium chloride dihydrate (Lima & Foguel, 2009 ▸)	7.5	293	11.6/29.5	2.21
4pvm	None	2.15 *M* sodium malonate (Haupt *et al.*, 2014 ▸)	6.4	293	7.7/–	2.21
1tta	None	40% ammonium sulfate and 200 m*M* citrate buffer (Hamilton *et al.*, 1993 ▸)	5.3	295	6.1/6.1	2.25
4tlt	None	10 m*M* sodium acetate, 100 m*M* KCl, 10 m*M* EDTA (Saelices *et al.*, 2015 ▸)	–	–	9.7/26.7	1.95
8ve2	None	18.75 µ*M* A2, 0.01%(*w*/*v*) BOG and 1.25%(*v*/*v*) DMSO (Basanta *et al.*, 2025 ▸)	7.6	RT	9.9/11.7	– (EM)
3tct	Tafamidis	1.395 *M* sodium citrate, 3.5%(*v*/*v*) glycerol (Bulawa *et al.*, 2012 ▸)	5.5	298	7.3/5.6	2.12
8aww	Tafamidis	50 mmol dm^−3^ MES–NaOH, 0.1 mol dm^−3^ NaCl, 5 mmol dm^−3^ DTT, 1 mmol dm^−3^ protease inhibitors (Cerofolini *et al.*, 2023 ▸); cryoEM	6.5	293	6.7/5.8	2.34
4d7b	Tolcapone	25% PEG 400, 200 m*M* calcium chloride, 100 m*M* HEPES (Sant’Anna *et al.*, 2016 ▸)	7	291	8.9/6.6	2.18
