# Supplementary material for: Differentiating the solution structures and stability of transthyretin tetramer complexed with tolcapone and tafamidis using SEC-SWAXS and NMR
Source: J Appl Crystallogr. 2025 Jul 8;58(Pt 4):1373–83. doi: 10.1107/S1600576725004716 (PMC12321038; doi:10.1107/S1600576725004716)
Supplement: Supplementary file 1 [file j-58-01373-sup1.pdf]

JOURNAL OF APPLIED CRYSTALLOGRAPHY

Volume 58 (2025)

Supporting information for article:

Differentiating the solution structures and stability of transthyretin tetramer complexed with tolcapone and tafamidis using SEC-SWAXS and NMR

Orion Shih, Yu-Chen Feng, Sashank Agrawal, Kuei-Fen Liao, Yi-Qi Yeh, Je-Wei Chang, Tsyr-Yan Yu and U-Ser Jeng

S1. TROSY-HSQC spectra of Apo-TTR

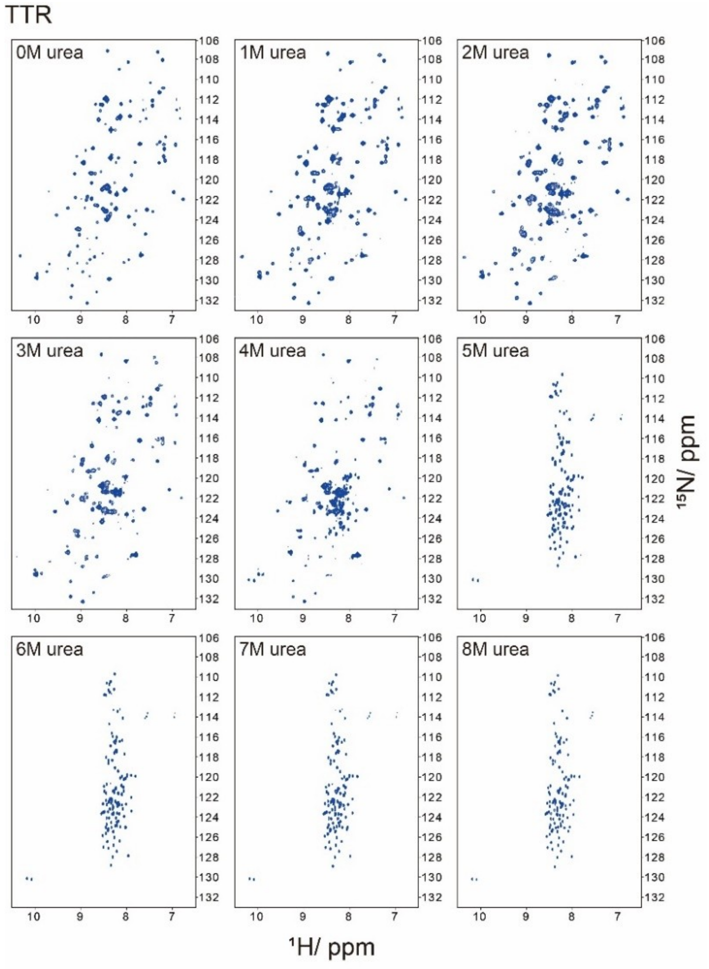

Figure S1. 2D [^{1}H,^{15}N]TROSY-HSQC spectra of Apo-TTR under different urea concentrations at 25 °C.

S2. Comparison of SWAXS data of TTR without and with ligand binding

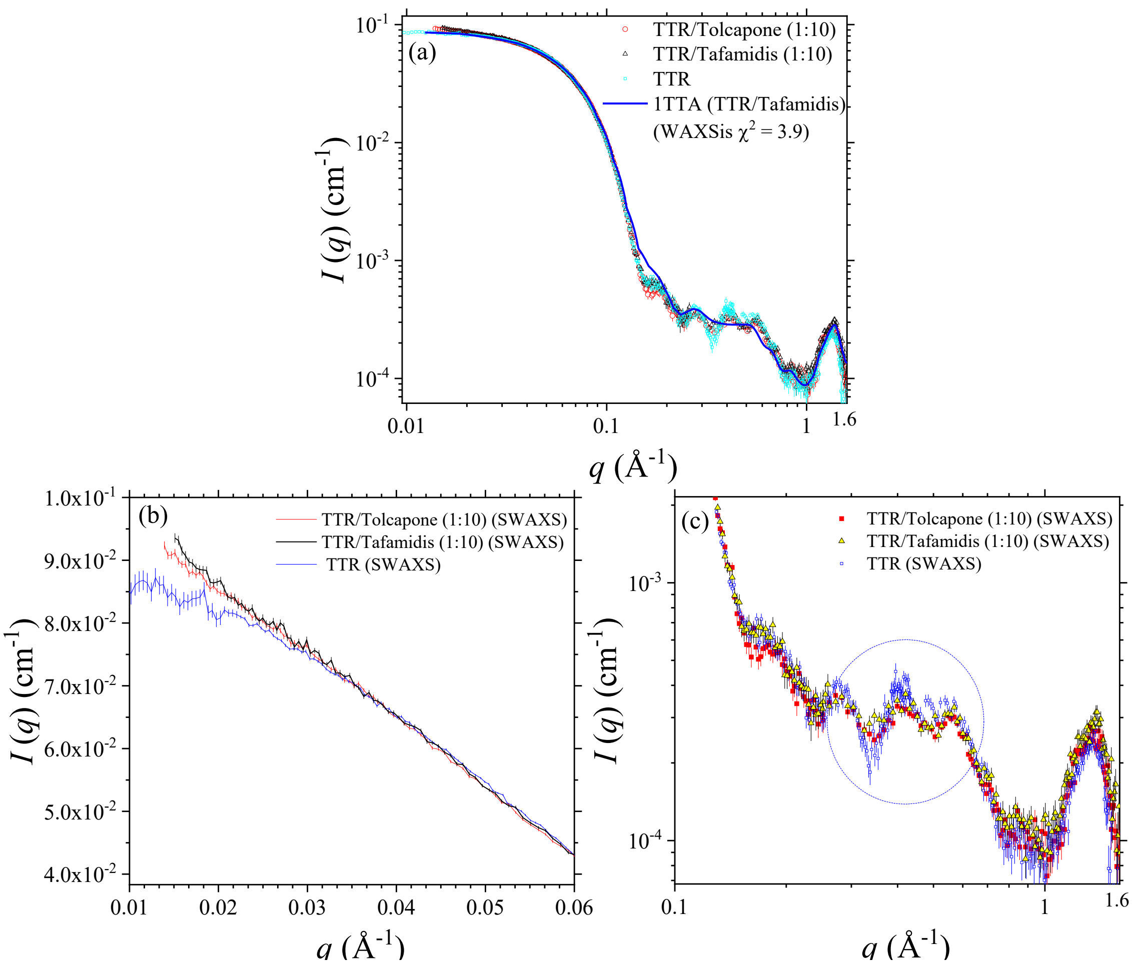

Figure S2. Comparisons of the SWAXS data for the Apo-TTR and TTR solutions with tolcapone or tafamidis (1:10 molar ratio, R = 10) in the (a) the full q-range, (b) low-q region, and (c) high-q region, measured at 37 °C. The circle in (c) marks the reduced intensity in the ligand-bounded cases, likely due to the ligand binding to the two empty T-4 sites, leading to a decreased scattering contrast of the T4 site pore of the TTR. Note that the R_g value of the Apo-TTR is about 1 Å smaller than that with either ligand, as described in the text. The data of TTR:Tafamidis 1TTA are fitted using the crystal structure 1TTA of Apo-TTR, resulting in a best-fitted χ^2 value of 3.9.

S3. SEC-SAXS profiles with 4M Urea measured at 37 °C(incubated at RT for 4 days)

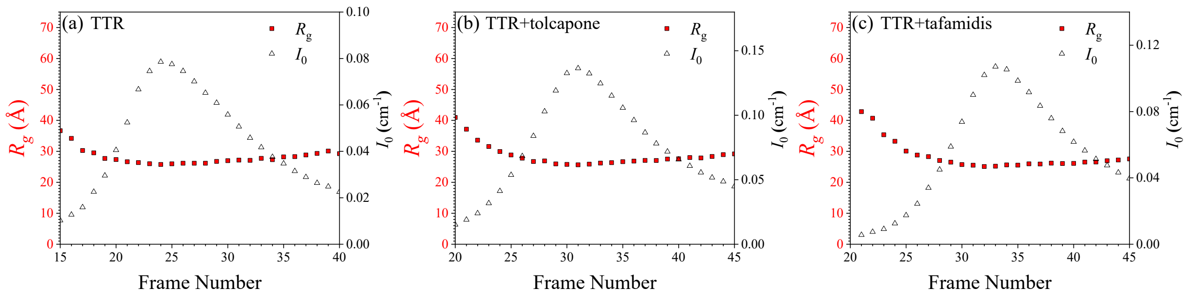

Figure S3. SEC elution profiles of SAXS I_o and R_g for (a) Apo TTR, (b) TTR with tolcapone, and (c) TTR with tafamidis in solutions of 4 M urea measured at 37 °C.

S4. SWAXS data for ligand-TTR solutions of 2:1 mixing ratio (*R* = 2)

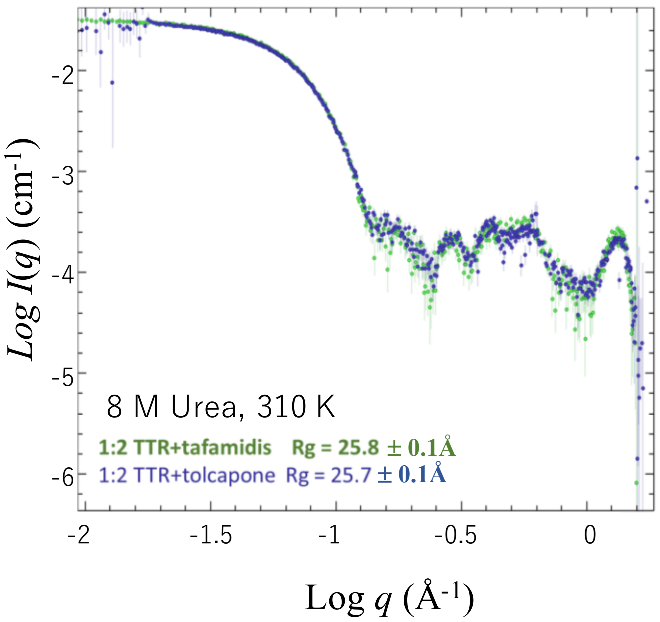

Figure S4. SWAXS data of the two incubated TTR solutions mixed with tolcapone and tafamidis at *R* = 2 in 8 M urea at 310 K. The well-overlapped SWAXS profiles and the close R_g values (as indicated) reveal that both compounds, with *R* = 2, can equally stabilize TTR in the 8 M urea-unfolding environment of a same incubation process; note that all the local structural features can be reserved largely, as revealed by the similar high-*q* scattering features compared to that without urea shown in Figure 2.

S5. Incubation time effect at 8 M urea and *R* = 10

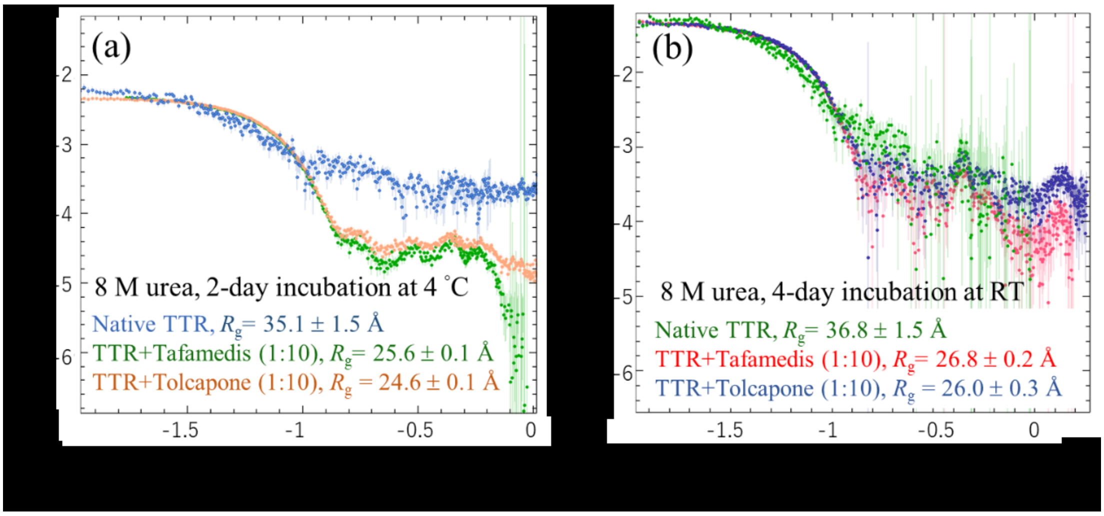

Figure S5. SWAXS data (relative intensity scale) of the TTR solutions without and with ligand (tolcapone or tafamidis) at *R* = 10 in 8 M urea, with the samples incubated (a) at 4 °C for 2 days and (b) RT for 4 days. The corresponding R_g values extracted from the SWAXS data are indicated.

S6. Scattering contribution of ligand binding

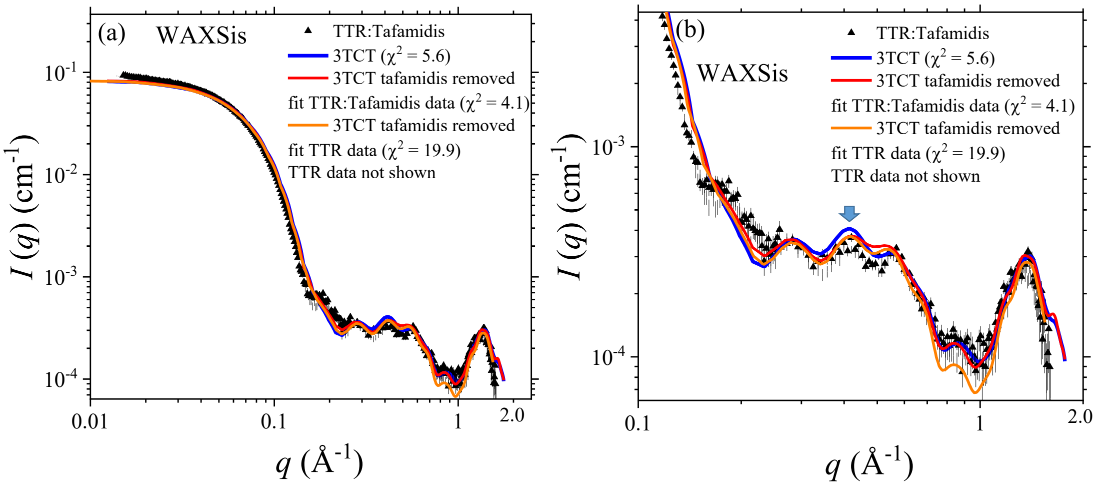

Figure S6. (a) Comparison of WAXSis fitting curves to the SWAXS data of the TTR:Tafamidis and native TTR (not shown), using the 3TCT crystal structure and 3TCT with the two tafamidis molecules removed. (b) Enlarged view focusing on the high-*q* region. The arrow indicates a slight intensity suppression in the SWAXS data fitting profile using the 3TCT crystal structure with the two ligands removed.

S7. Example of protein unfolding effect on sample elution time

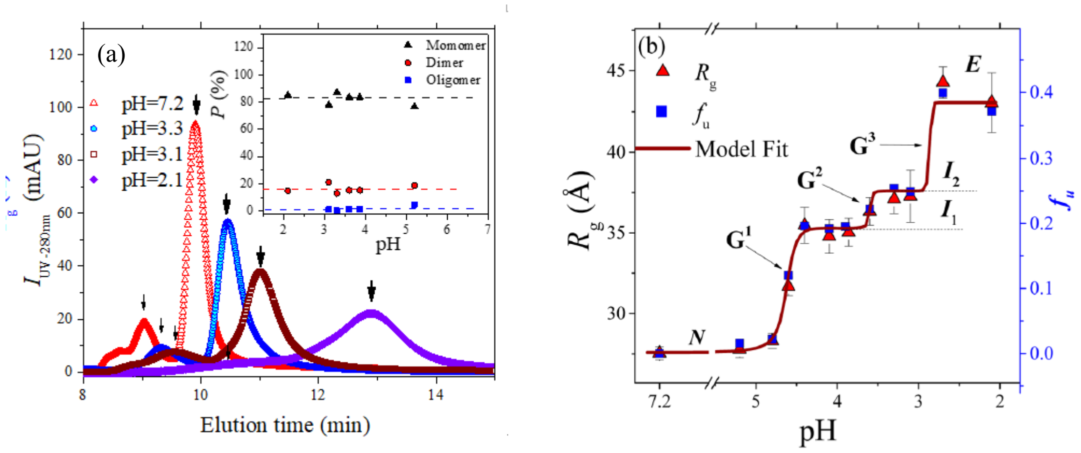

Figure S7. (a) Representative chromatograms for bovine serum albumin (BSA) in the solutions at pH 7.2 - 2.1. Note that the protein elution peak is increasingly delayed with respect to that of the folded protein of a compact conformation. The monomer and dimer elution peaks are marked with thick and thin arrows, respectively. Inset shows the molar fractions *P* extracted from the corresponding integrated peak areas of the species indicated. (b) The corresponding R_g extracted. (data are adopted from the publication of Yeh, et al., 2017).

S8. SAXS Data Collection Table (Trewhella et al. 2017)

Table S1. SAXS data collection parameters and processing details

Sample name	TTR	TTR with over-saturated tolcapone (*R* = 10)	TTR with over-saturated tafamidis (*R* = 10)	TTR in 8 M urea at 37 ℃ (P1)	TTR in 8 M urea at 37 ℃ (P2)	TTR-tolcapone (*R* = 10) in 8 M urea at 37 ℃	TTR-tafamidis (*R* = 10) in 8 M urea at 37 ℃	TTR-tafamidis (*R* = 1) in 8 M urea at 37 ℃
Sample details								
Calculated molecular weight	55.0 kDa	55.6 kDa	55.7 kDa	110.1 kDa	165.1 kDa	55.6 kDa	55.7 kDa	55.4 kDa
SEC column	Agilent Bio SEC-3 LC Column (pore size 300 Å)							
Number of frames used for data analysis	8	5	3	3	6	3	5	8
Injected volume (μL)	100							
Loading concentration (mg/mL)	5	8	8	8	8	8	8	8
SEC buffer	20 mM Tris 50 mM NaCl pH 7.0	20 mM Tris 50 mM NaCl pH 7.0	20 mM Tris 50 mM NaCl pH 7.0	20 mM Tris 50 mM NaCl in 8M urea pH 7.0	20 mM Tris 50 mM NaCl in 8 M urea pH 7.0	20 mM Tris 50 mM NaCl in 8 M urea pH 7.0	20 mM Tris 50 mM NaCl in 8 M urea pH 7.0	20 mM Tris 50 mM NaCl in 8 M urea pH 7.0
Temperature (°C)	26	26	26	37	37	37	37	37
Flow rate (mL/min)	0.35							
SAXS data collection parameters								
Instrument	TPS 13A BioSWAXS beamline of the National Synchrotron Radiation Research Center							
Wavelength (Å)	0.8265							
q range ($Å^{-1}$)	0.007 – 0.6 (SAXS); 0.4 – 2.4 (WAXS)							
Sample-to-detector distance (m)	2.5 (Eiger X 9M) 0.288 (Eiger X 1M)							

Exposure time	2 sec/frame							
Detector(s)	Eiger X 9M (SAXS) and X 1M (WAXS) detectors, both in vacuum							
Flux (photons/s)	~1x10^{12}							
Beam size (μm)	300(H)x225(V)							
Sample configuration	2.0 mm diameter quartz capillary							
Absolute scaling method	Scaling to absolute water scattering intensity							
Normalization	Accumulated monitor counts of the incident X-ray flux over the sample measuring time.							
Structural parameters								
R_g (Å) [from p(r)]	24.5	25.0	25.1	37.0	52.0	26.1	26.1	NA
R_g (Å) [from Guinier]	24.3±0.1	25.2±0.1	25.2±0.1	36.8±1.5	51.8±1.9	26.0±0.5	26.8±0.5	28.2±0.3
D_{max} (Å)	75.0	86.0	90.2	120.0	161.5	77.0	77.5	NA
MW determined from size & shape (kDa)	58.7	63.5	59.3	90.6	195.1	62.2	71.9	NA
MW determined from V_c (kDa)	54.0	54.3	53.3	60.8	118.2	53.7	59.5	NA
Software employed								
SAXS data reduction and data processing	TPS 13ASWAXS Data Reduction Kit (Ver. 4.88)<br>PRIMUS (ATSAS 3.2.1)							
Computation of model intensities	CRYSOL (ATSAS 3.2.1)<br>WAXSiS							

Reference.

(S1) Trewhella, J., Duff, A. P., Durand, D., Gabel, F., Guss, J M., Hendrickson, W. A., Hura, G. L, Jacques, D. A, Kirby, N. M., Kwan, A. H., Pérez, J., Pollack, L., Ryan, T. M., Sali, A., Schneidman-Duhovny, D., Schwede, T., Svergun, D. I., Sugiyama, M., Tainer, J. A., Vachette, P., Westbrook, J., Whitten, A. E., (2017), *Acta Crystallogr. D Struct. Biol.* 73, 710-728.

(S2) Yeh, Y. Q., Liao, K. F., Shih, O., Shiu, Y. J., Wu, W. R., Su, C. J., Lin, P. C. & Jeng, U. S. (2017). *J. Phys. Chem. Lett.* 8, 470-477
